# Poverty and large family size are pivotal risk factors for child marriage in Ethiopia: evidence from a matched case-control study

**DOI:** 10.3389/fsoc.2025.1544169

**Published:** 2025-05-23

**Authors:** Shiferaw Gelchu Adola, Dessalegn Wirtu, Merga Dheresa

**Affiliations:** ^1^Department of Nursing, Institute of Health, Bule Hora University, Bule Hora, Ethiopia; ^2^Department of Public Health, College of Health and Medical Science, Wollega University, Nekemte, Ethiopia; ^3^School of Nursing and Midwifery, College of Health and Medical Sciences, Haramaya University, Harar, Ethiopia

**Keywords:** risk factors, child marriage, West Guji Zone, Southern Oromia, Ethiopia

## Abstract

**Background:**

Child marriage is a marriage carried out before the age of 18 years old which is influenced by numerous cultural, social, and economic factors, and it is a source of gender inequality, violence against women, and various maternal and child health problems. Therefore, this study aimed to identify risk factors of child marriage in the West Guji zone of southern Oromia, Ethiopia.

**Methods:**

A community-based 1:2 matched case-control study design was conducted from April 1 to May 5, 2024. The purposive sampling technique was used to select 120 pairs (120 cases and 240 controls). Age was selected as a matching variable, and for each case, two best-matched controls were selected. The questionnaires were administered through a face-to-face interview. Both binary and multivariable conditional logistic regressions were conducted to determine independent determinants of child marriage. In the multivariable model, statistical significance was established at *p* < 0.05. The strength of the association was reported by the matched adjusted odds ratio (mAOR) with 95% CI.

**Results:**

Women from medium family size (4–6 members) were nearly four times more likely to be married early as compared to women from small family size (≤ 3 family members) (mAOR: 3.87, 95% CI = 1.56–9.55). The odds of early marriage were five-fold greater among women from larger families (mAOR: 5.09, 95% CI = 1.53–16.90). The odds of being married younger than 18 years were nearly three times greater among women who were not aware of the legal age of marriage (mAOR: 2.92, 95% CI = 1.27–6.68). Women whose marriage decision was made by others were two times more likely to be married early (mAOR: 2.47, 95 CI = 1.30–4.71). The risk of marriage under 18 years of age was seven-fold greater among women from the poor parental wealth category than among those from the rich category (mAOR: 7.65, 95%CI = 2.48–13.07).

**Conclusion:**

Poor family wealth statuses, larger parental family size, not knowing the legal marital age, and marriage decision by others, were risk factors for child marriage. As a result, to stop child marriage in rural Ethiopia, policies and strategies that take into account these factors should be developed and put into practice.

## Introduction

Marriage is a process in which the legitimate relationship of husband and wife is established and the legality of the union is granted according to the law of each country, whether through civil, religious, or customary (Haarr and Duncan, 2023). A child is any individual under the age of 18 (Assembly, 1989; Charter, 1990). In the same way, child marriage is a marriage that occurs when one or both of the partners are under the age of eighteen, which is viewed as a harmful prejudice that restricts children's freedom (UNICEF, 2014; Fernandes and Ambewadikar, 2022). It is a global human rights issue that promotes gender inequality and significantly harms girls (Koski et al., 2023). High-risk maternal and child health outcomes, including physical sickness, high-risk pregnancy and delivery, emotional distress, low birth weight, malnutrition, infant and child diarrhea, and death, are associated with child marriage (Yoosefi Lebni et al., 2023; Raj et al., 2010).

Globally, ~640 million women and girls alive today were married before 18 years old. South Asia and sub-Saharan Africa account for more than two-thirds of child marriages (UNICEF, 2023). In sub-Saharan Africa, nearly one in three girls were married before turning 18 years old. Ethiopia is one of the top five countries in sub-Saharan Africa in terms of child marriage (Fund UNCS, 2018). Child marriage is a public health and social problem in Ethiopia where more than half (56.34%) of girls were married before the age of 18 years (Gebeyehu et al., 2023).

Although child marriage occurs worldwide, it is exacerbated by rural living, poverty, conflict, gender inequality, lack of access to education, and underdeveloped places with limited access to healthcare (Raj, 2010; Binu et al., 2022). Additional risk factors for child marriage include the husband's education, employment, religion, ethnicity, family size, marriage decision by the parents, lack of other options for girls rather than marriage, fear of premarital sexual affairs, social norms, and lack of legal pressure against child marriage, which are all risk factors that differ from place to place (Gebeyehu et al., 2023; Pourtaheri et al., 2023; Misunas et al., 2021; Wilkinson et al., 2024).

Generally, child marriage is a prevalent practice among rural girls (Yogi, 2020; Ghrayeb et al., 2015). Ethiopian girls remain at risk of child marriage because nearly 80% of the population resides in rural areas (MoH, 2021). According to studies conducted in Ethiopia, rural women are more likely to marry at an early age than urban women (Abdumalik et al., 2018; Aychiluhm et al., 2021; Workineh et al., 2015; Lami et al., 2023). Nevertheless, none of the studies reported risk factors for marriage at an early age among rural women.

Basically, the issue of child marriage concerns governments, international organizations, and civic societies (Koski et al., 2023). The United Nations Sustainable Development Goal (SDG) states that child marriage must be eradicated by 2030 to guarantee gender equality (Nations, 2015). Article 21 of the African Charter on the Rights and Welfare of the Child (ARWC) and Article 34 of the Convention on the Rights of the Child (CRC) mentioned the protection of children from detrimental social and cultural practices (Charter, 1990; Assembly, 1989). The Ethiopian government is also committed to ending gender-based violence, including child marriage, by 2025 (MoH, 2021).

Although the susceptibility of rural girls to child marriage has been reported in Ethiopia, the potential risk factors have been insufficiently addressed, suggesting the need for further study. Therefore, this study aimed to identify determinants of child marriage among rural women in southern Oromia to generate evidence that is relevant to support interventional strategies against child marriage.

## Methods

### Study setting and period

This community-based matched case-control study was carried out in the West Guji Zone, Southern Oromia. The zone is located 470 km toward the south of Ethiopian capital city, Addis Ababa. The Zone comprises 196 kebeles (the lower-level administrative unit) and 10 administrative districts (9 rural districts and 1 urban district). The healthcare facilities in the zone that provide healthcare services to the local people and the surrounding area include one teaching hospital, three district hospitals, forty-three health centers, and 197 health posts.

According to a 2024 report of the West Guji Zone Administration Office, the zone had over 2.5 million inhabitants, of which 49.6% were women and 50.4% were men. In rural districts, there are 303,636 women of reproductive age. This study was conducted from April 1 to May 5, 2024.

### Study design

A community-based 1:2 matched case control study design was used to compare the risk factors of early marriage among women who were married before 18 years of age (cases) and those who were married after 18 years of age (controls).

### Study population

The study populations for this study were women of reproductive age (15–49 years) living in the West Guji Zone and who were available in their residential home during the study period.

### Cases

Cases defined as women who were married before turning 18 years old.

### Controls

Controls defined as women, those who were married after 18 years of age.

### Inclusion criteria

The inclusion criteria for cases were being married before 18 years of age, living in the west Guji zone for more than 6 months (permanent inhabitants), having no serious health problems that interfered with communication during the interview, and being volunteers to participate in the study. Similarly, the inclusion criteria for controls were being married after the age of 18 years, being permanent residents of the West Guji Zone, having no serious health problems that hindered the interview, having interest in taking part in the study voluntarily, and living in a similar village with the cases.

### Matching

Women who were married before 18 years of age were matched 1:2 for current age (±2 years) and places of residence with women who were married after 18 years of age. Current age of the participants and places of residence (neighborhoods) selected as matching variables with the assumption that they are a powerful confounders.

### Study variables

The dependent variable was having ever married before turning 18 years. The independent variables include; sociodemographic factors, service-related characteristics, personal related variables and context-pertaining characteristics.

### Sample size determination

An online calculator was used to determine the sample size for a matched case-control study design (Stevenson et al., 2015), taking into account a 95% confidence level, 80% power, a 2:1 control-to-case ratio, a correlation coefficient (*r*) for exposure between matched cases and controls = 0.2, and different sample sizes calculated for various risk factors for early marriage. The maximum sample size was obtained from fathers' education level as a risk factor for marriage at an early age from the former a study conducted in the Amhara region, where the proportion of exposure among controls was 17.69% and odds ratio (OR = 2.3) (Bezie and Addisu, 2019). The final sample size for this study was 120 cases and 240 controls, or 360 participants, after adding a 10% nonresponse rate. All cases who satisfied the inclusion criteria were included in the sample purposively until the required number of samples was obtained. For each case, two controls that were best matched were identified.

### Sampling techniques

A purposive sampling technique was used to select the cases and controls. For each case, two best- matched controls who met the inclusion criteria were selected purposively until the required number of samples was obtained.

### Data collection tools and quality control

The questionnaires for data collection were written in English, translated into Afan Oromo, and translated back to English for consistency. Two language specialists were invited to verify the questions for grammatical errors and assess the questionnaires' coverage (breadth) and content (depth). Prior to the actual data collection, the tools were pretested on 5% of the total study subjects in the East Guji Zone who were not included in the actual data analysis. Its consistency, clarity, and logical sufficiency were amended in light of the findings, and the interview's completion duration was modified. In addition, the validity of the instruments was statistically evaluated for internal consistency (reliability) via Cronbach's alpha reliability scale. The questionnaires were administered through a face-to-face interview technique.

For 2 days, supervisors and data collectors (health extension staff) received training on the study's instrument, data collection process, informed consent, and information confidentiality. After data collection, supervisors and the principal investigator checked the completed questionnaires daily for completeness.

### Data analysis

The data were entered into EpiData version 4.4.3.1 to minimize data entry errors and then exported them to Stata version 14 for analysis. An appropriate data entry method for matched case- controls was applied on the basis of the matching variable of current age in which two matched controls were entered for one case. The data was entered concurrently following two matched controls per case. In this research, both descriptive and inferential statistics were used. For numerical continuous variables, data summary measure, the mean was calculated and the proportion was used to display the results for categorical variables. To examine the relationships between risk factors and outcome variables, conditional logistic regression analyses of both binary and multivariable models were performed. In the bivariate analysis, significant variables at *p* < 0.2 were selected as candidates for multivariable conditional logistic regression. Statistical significance was established at *p* < 0.05. The strength of the association was assessed by the matched adjusted odds ratio (mAOR) with 95% CI. Model fitness was checked by the post-estimation command (Hosmer and Lemeshow test) in Stata by running the estat gof commend. As a result, the Hosmer and Lemeshow chi-square *p*-value was >0.05, indicating the model's fitness. The study's report was written using the STROBE case-control checklist (Von Elm et al., 2007).

## Results

### Sociodemographic characteristics of the cases and controls

In total, 360 married women of reproductive age participated in this study. A total of 120 matched pairs, 120 women who were married before turning 18 years old (cases) and 240 women who were married after 18 years of age (controls), were interviewed. The mean age of the cases and controls at the time of interviews was 28.65 ± (4.5 SD) and 28 ± (4.3 SD) years, respectively. The minimum and maximum ages of the cases were 20 and 40 years, whereas the minimum and maximum ages of the controls were 20 and 41 years old. The majority of 57 (47.5%) cases and 107 (44.6%) controls were between 24 and 29 years of age; were as almost similar proportion of 11.7% cases and 12.1% of the controls were in an age category equal to or >35 years.

With respect to the marital status of the participants, a comparable proportion of cases (95%) and controls (95.4%) were in unions, whereas (4.2%) of the cases and (3.8%) of the controls were divorced. The majority of the cases 99 (82%) and the controls 206 (85.8%) were from the Oromo ethnic group. Sixty-eight (56.7%) cases and 119 (49.6%) controls were protestant religion followers. More than half of the participants' parents had formal education, and very few of them had secondary and above. With respect to participants' education level, one-third of the cases 44 (36.7%) and controls 84 (35%) had no formal education, and only 13 (10.8%) cases and 65 (27.1%) controls learned secondary school and above.

In terms of participants' occupation, almost half of the cases (49.2%) and controls (52.1%) were housewives. With respect to the participants' parents family size, (60%) of the cases and (43.8%) of the controls parents had medium family size (4–6 members) and (23.3%) of the cases and 16.7% of the controls parents had a larger family size of seven and above members. Regarding the participants' parental wealth, half of the cases 62 (51.6%) and 54 (22.5%) of the controls were from poor family and 20 (16.7%) of the cases and 100 (41.7%) of the controls were from richer parental wealth categories (Table 1).

**Table 1:** Sociodemographic characteristics of women married before (cases) and after 18 years old (controls) in West Guji zone, Southern Ethiopia, 2024.

**Characteristics**	**Category**	**Cases (%)**	**Controls (%)**
		***N* = 120**	***N* = 240**
Participants' current age	20–24	19 (15.8)	41 (17.1)
	25–29	57 (47.5)	107 (44.6)
	30–34	30 (25)	63 (26.2)
	≥35	14 (11.7)	29 (12.1)
Marital status of participants' parents	Married	103 (85.8)	220 (91.7)
	Divorced	6 (5)	8 (3.3%)
	Widowed	11 (9.2)	12 (5)
Marital status of the participants	Marriedsss	114 (95)	229 (95.4)
	Divorced	5 (4.2)	9 (3.8)
	Widowed	1 (0.8)	2 (0.8)
Ethnicity	Oromo	99 (82.5)	206 (85.8)
	Gedio	13 (10.8)	22 (9.2)
	Others (Amhara Burji)	8 (6.7)	14 (5)
Religion	Orthodox	14 (11.7)	54 (22.5)
	Muslim	28 (23.3)	40 (16.7)
	Protestant	68 (56.7)	119 (49.6)
	Waqefata	10 (8.3)	27 (11.2)
Father education level	Had no formal	89 (74.2)	137 (57.1)
	Primary (1–8)	28 (23.3)	66 (27.5)
	Secondary (9–12) and above	3 (2.5)	37 (15.4)
Mother education level	Had no formal	113 (94.2)	197 (82.1)
	Primary (1–8)	7 (5.8)	28 (11.7)
	Secondary (9–12) and above	15 (6.2)	0 (0%)
Participant education level	Had no formal	44 (36.7)	84 (35)
	Primary (1–8)	63 (52.5)	91 (37.9)
	Secondary (9–12) and above	13 (10.8)	65 (27.1)
Participants occupation	Farmer	18 (15)	43 (17.9)
	Housewife	59 (49.2)	125 (52.1)
	Merchant	29 (24.2)	34 (14.2)
	Others (daily worker, gov't employee)	14 (11.6)	38 (15.8)
Parents family size	1–3	20 (16.7)	95 (39.6)
	4–6	73 (60)	105 (43.8)
	≥7	27 (23.3)	40 (16.7)
Parents' wealth	Poor	62 (51.6)	54 (22.5)
	Middle	38 (31.7)	86 (35.8)
	Rich	20 (16.7)	100 (41.7)

### Service-related attributes

The majority of the study participants (80%) of the cases and (84.6%) of the controls had media exposure and radio was the main media they followed. One hundred one (84.2%) cases and 191 (79.6%) controls reported a lack of girls supporting agencies in their vicinity. Nearly one-third of the cases (29.2%) and 89 (37.1%) of the controls reported an absence of legal action against early marriage nuisance in their locality. The majority of cases 114 (95%) and controls 112 (88.3%) reported a lack of job opportunities for females in their surroundings (Table 2).

**Table 2:** Service-related attributes of women married before and after 18 years old in West Guji zone, Southern Ethiopia, 2024.

**Variables**	**Category**	**Cases (%)**	**Controls (%)**
		***N* = 120**	***N* = 240**
Exposure to media	No	24 (20)	37 (15.4)
	Yes	96 (80)	203 (84.6)
The type of media exposed	Radio	80 (83.3)	118 (58.1)
	TV	16 (16.7)	85 (41.9)
Availability of girls' supporting agencies	No	101 (84.2)	191 (79.6)
	Yes	19 (15.8)	49 (20.4)
Legal punishment against perpetrators of early marriage	No	35 (29.2)	89 (37.1)
	Yes	85 (70.8)	151 (62.9)
Job opportunities for females in your locality	No	114 (95)	112 (88.3)
	Yes	6 (5)	28 (11.7)

### Personal related characteristics

One hundred eleven cases (92.5%) and 219 (91.2%) controls thought that sexual intercourse before marriage was incorrect whereas nine cases (7.5%) and 21 (8.8%) of controls recognized it as a correct. Forty-four (36.7%) of cases and 126 (52.5%) believed that sexual intercourse before marriage was the causes of early marriage, whereas more than half of cases 76 (63.3%) believed that sexual intercourse before marriage did not lead to early marriage. The majority of the cases and controls lived with their parents before their marriage, whereas few of them lived alone. Ninety-six (80%) cases and 214 89.2%) controls were aware the legal age of marriage and 24 (20%) cases and 26 (10.8) controls did not know the legal marital age (Table 3).

**Table 3:** Personal related characteristics of women married before and after 18 years old in West Guji zone, Southern Ethiopia, 2024.

**Variables**	**Category**	**Cases (%)**	**Controls (%)**
		***N* = 120**	***N* = 240**
Your opinion on having sex before getting married	Not correct	111 (92.5)	219 (91.2)
	Correct	9 (7.5)	21 (8.8)
Do you believe that having sex before marriage leads to early marriage?	No	76 (63.3)	114 (47.5)
	Yes	44 (36.7)	126 (52.5)
Live with before marriage	Parent	77 (64.2)	183 (76.2)
	Relative	32 (26.6)	35 (14.6)
	Alone	11 (9.2)	22 (9.2)
Know the legal age of marriage	No	24 (20)	26 (10.8)
	Yes	96 (80)	214 (89.2)

### Context-related factors

One hundred seven (89.1%) cases and 190 (79.2%) controls reported that the appearance of signs of puberty was viewed as criterion for girls being adequate for marriage. Nearly two-thirds of the cases and controls mentioned the custom of girls marrying before 18 years of age in their community.

Thirty-six (39.6%) of the cases reported that reason for early marriage was; to protect girls' virginity up to marriage, to avoid premarital sexual intercourse, and 12 (13.2%) of them reported lack other options for girls, whereas 43 (27.2%) controls mentioned protecting girls' virginity, 66 (41.8%) of them stated that to avoiding premarital sexual intercourse, and 28 (17.7%) said that a lack of other options for girls was the main reason for girls' marriage before 18 years of age.

Almost all of the study participants reported that there was fear related to girls' age in their community. The reasons behind these findings were that girls may face difficulty marrying if they are older, for the reputation of their family, and to avoid stigma related to girls' age; 47 (40.2%), 59 (50.4%), and 11 (9.4%), respectively, among cases, whereas marriage difficulty, family reputation, and stigma related to older age, 122 (53.5%), 73 (32%) and 33 (14.5%) among the controls. Nearly half 68 (56.7%) of the cases and two-thirds 173 (72.1%) of the controls decided their marriage on their own.

For 52 (43.3%) cases and 67 (27.9%) controls, marital decisions were made by others. Almost half of the cases and controls reported the presence of security problems in their surroundings. The majority (83.3%) of the cases and 211 (87.9) controls mentioned the absence of conflict within their family (Table 4).

**Table 4:** Context-related factors of women married before and after 18 years old in the West Guji zone, Southern Ethiopia, 2024.

**Characteristics**	**Category**	**Cases (%)**	**Controls (%)**
		***N* = 120**	***N* = 240**
The criterion for girl's reached for marriage in your vicinity	Signs of puberty (breast enlargement, menses)	107 (89.1)	190 (79.2)
	Age >18 years old	11 (9.2)	38 (15.8)
	Finished school or graduate	2 (1.7)	12 (5)
The custom of girls marriage age <18 years old in your locality	No	29 (24.2)	82 (34.2)
	Yes	91 (75.8)	158 (65.8)
Reason for marriage age <18 years old in your vicinity	To protect girls' virginity	36 (39.6)	43 (27.2)
	To avoid premarital affairs	36 (39.6)	66 (41.8)
	To strengthen inter-family relationship	7 (7.7)	21 (13.3)
	Lack of other options for girls	12 (13.2)	28 (17.7)
Is there fear in your area if girls grow older?	No	3 (2.5)	12 (5)
	Yes	117 (97.5)	228 (95)
Reason for fear	Face difficulty to marry	47 (40.2)	122 (53.5)
	For family reputation	59 (50.4)	73 (32)
	Stigma related to older age	11 (9.4)	33 (14.5)
Who decided your marriage?	Not self (father, mother, relative)	52 (43.3)	67 (27.9)
	Self	68 (56.7)	173 (72.1)
Honor for girls related to girls' virginity	No	2 (1.7)	35 (14.6)
	Yes	118 (98.3)	205 (85.4)
Honor for parents related to girls' virginity	No	11 (8.2)	35 (14.6)
	Yes	109 (90.8)	205 (85.4)
Security problem in your vicinity	No	65 (54.2)	125 (52.1)
	Yes	55 (45.8)	115 (47.9)
Conflict within family	No	100 (83.3)	211 (87.9)
	Yes	20 (16.7)	29 (12.1)

### Determinants of child marriage

A bivariate conditional logistic regression analysis was carried out to select variables for multivariable conditional logistic regression, and 8 variables were significant at *p* < 0.2. These variables are fathers' level of education, respondents' level of education, parents' family size, and media exposure, believing that sex before marriage causes early marriage, awareness of the legal age of marriage, marriage decision-making, and parents' wealth status.

In the multivariable conditional logistic regression, four variables were significant at *p* < 0.05. Parental family sizes, awareness of the legal age of marriage, marriage decision making, and parents' wealth status.

Women from medium family size (4–6 family members) were nearly four times more likely to be married early as compared to women from small family size (1–3 family members) (mAOR = 3.87, 95% CI = 1.56–9.55). Similarly, odds of early marriage were five-fold greater among women from larger family size (≥7 family members) (mAOR; 5.09, 95% CI = 1.53–16.90) compare to small family size. The likelihood of marriage being <18 years was nearly three times greater among women who were not aware of the legal age of marriage (mAOR = 2.92, 95% CI = 1.27–6.68) than among those who were aware of the legal age of marriage.

Women whose marriage decision was made by others were two times more likely to be married early than those who decided their marriage on their own (mAOR = 2.47, 95% CI = 1.30–4.71). Parental economic status was risk factor for child marriage. The risk of child marriage was seven-fold greater among women from the poor parental wealth category than among those from the rich category (mAOR = 7.65, 95% CI = 2.48–13.07; Table 5).

**Table 5:** Risk factors of child marriage of women married before and after 18 years old in West Guji zone, Southern Ethiopia, 2024.

**Variables**	**Category**	**EM/cases**	**No EM/controls**	**mCOR (95% CI)**	**mAOR (95% CI)**	***p*-value**
		***N* = 120**	***N* = 240**			
		***N* (%)**	***N* (%)**			
Father education level	Non formal	89 (74.2)	137 (57.1)	8.56 (2.57–29.05)	3.01 (0.60–15.03)	0.179
	Primary	28 (23.3)	66 (27.5)	6.00 (1.68–21.33)	3.06 (0.58–16.14)	0.188
	Sec and above	3 (2.5)	37 (15.4)	1	1	
Participant education level	Non formal	44 (36.7)	84 (35)	2.50 (1.23–5.08)	1.48 (0.57–3.75)	0.436
	Primary	63 (52.5)	91 (37.9)	3.63 (1.83–7.21)	2.09 (0.89–4.91)	0.091
	Sec and above	13 (10.8)	65 (27.1)	1	1	
Parents family size	1–3	20 (16.7)	95 (39.6)	1	1	
	4-6	73 (60)	105 (43.8)	5.41 (2.73–10.69)	3.87 (1.56–9.55)	0.003^*^
	≥7	27 (23.3)	40 (16.7)	6.79 (2.84–16.23)	5.09 (1.53–16.90)	0.008^*^
Media type	Radio	80 (83.3)	118 (58.1)	3.83 (2.02–6.90)	0.33 (0.06–1.82)	0.206
	Tv	16 (16.7)	85 (41.9)	1	1	
Believed sex before marriage cause early marriage	No	76 (63.3)	114 (47.5)	2.06 (1.29–3.30)	0.78 (0.35–1.74)	0.554
	Yes	44 (36.7)	126 (52.5)	1	1	
Awareness of legal age	No	24 (20)	26 (10.8)	2.00 (1.09–3.66)	2.92 (1.27–6.68)	0.011^*^
	Yes	96 (80)	214 (89.2)	1	1	
Marriage decision maker	Not self	52 (43.3)	67 (27.9)	1.94 (1.23–3.09)	2.47 (1.30–4.71)	0.006^*^
	Self	68 (56.7)	173 (72.1)	1	1	
Parents wealth status	Poor	62 (51.6)	54 (22.5)	6.54 (3.48–12.30)	7.65 (2.48–13.07)	0.005^*^
	Middle	38 (31.7)	86 (35.8)	2.34 (1.27–4.33)	3.88 (0.7–20.97)	0.114
	Rich	20 (16.7)	100 (41.7)	1	1	

## Discussion

The aim of this study was to generate the most relevant evidence to support interventional strategies against the practice of child marriage via a matched case-control study design. We confirmed four major risk factors for child marriage in the West Guji Zone, including the size of the parents' family, knowing the legal age of the marriage, marriage decision-making, and the wealth status of the girls' family. As a result, these findings will aid in the development of context-based strategies to prevent early marriage. One risk factor for child marriage among girls is the size of the parent's family. Households that have larger family members are in favor of early marriage (Hamed and Yousef, 2017; Pourtaheri et al., 2023). Girls from families with more than five daughters and sons are more likely to marry at an early age (Ali et al., 2014). Similar to the findings of the present study, women from families with four or more members were more likely to marry early than women with families fewer than four members. Parents in many parts of the world encourage their daughters to marry while still at a young age, hoping that the marriage will ease the strain of raising girls and assisting them socially and financially (UNICEF, 2005). The majority of the participants' parents had a family size of four or above in the current study. This finding has implications for policymakers because limiting the number of children supports the effort to control child marriage.

In line with previous studies (Tekile et al., 2020; Lami et al., 2023; Karki et al., 2024), the present study also revealed that not knowing the legal age of marriage was a factor significantly associated with child marriage. This is because the appearance of signs of puberty is viewed as a criterion for girls ready for marriage, in addition to the inadequacy of legal action against perpetrators of early marriage in the study area. Although the legal age of marriage is 18 years in Ethiopia (MoH, 2021).

Marital decision-making was found to be an independent predictor of child marriage. Women whose marriage decision was made by others were two times more likely to be married early than those who decided their marriage on their own. This finding was supported by earlier studies (Rahman and Kabir, 2005; Lami et al., 2023; Gebeyehu et al., 2023; Aychiluhm et al., 2021; Alem et al., 2020). Parental decision -making concerning when to marry is common in Ethiopian culture (Assefa et al., 2005). The reasons for early marriage in the study area include protecting girls' virginity, avoiding premarital sexual intercourse, lacking other options for girls, and strengthening family relationships through marital ties.

Poverty is the major influencing factor contributing to girls' child marriage in low and middle-income countries (Lebni et al., 2020; Madut, 2020; Mpilambo et al., 2017; Pourtaheri et al., 2024; Chenge and Maunganidze, 2017). Moreover, previous studies conducted in Ethiopia demonstrated that poor economic family status was a key factor in determining child marriage practices (Bezie and Addisu, 2019; Aychiluhm et al., 2021; Workineh et al., 2015). In a similar vein, the present study confirmed that the risk of early marriage was seven-fold greater among women from the poor parental wealth category than among those from to the rich. This is because parents in developing nations push their daughters to marry at an early age for financial reasons (Kok et al., 2023). This suggests that all relevant bodies strive to improve the financial status of low-income families and provide support for young girls.

Even though this study used a matched case-control study design to identify relevant risk factors for child marriage, cases and controls were selected by purposive sampling technique, which may limits the generalizability of the findings. Cases and controls selected on the basis of inclusion criteria rather than random sampling might introduce selection bias. The participant's age during marriage was asked retrospectively. Thus, there may be a possibility of recall bias.

## Conclusion

This study assessed different risk factors for child marriage. As a result, parental family size, awareness of the legal marital age, marriage decision-making, and parents' wealth status were identified as relevant risk factors for child marriage in the study area. In light of these findings, limiting the size of the family, improving the economic status of the community and financial support for adolescent girls, creating awareness on legal age of marriage and not involving girls' parents (father, mother) and relatives in marital decisions are recommended. Moreover, to stop child marriage in rural Ethiopia, policies and strategies that increase family wealth and limit the number of children that should be born ought to be developed and put into practice. In addition, providing training for girls, families, and communities on the harmful effects of child marriage to create awareness and keep girls at school by giving support to empower girls through education was recommended.

## Data Availability

The original contributions presented in the study are included in the article/supplementary material, further inquiries can be directed to the corresponding author.
